# Adhesive capsulitis of the shoulder: protocol for the adhesive capsulitis biomarker (AdCaB) study

**DOI:** 10.1186/s12891-019-2536-x

**Published:** 2019-04-05

**Authors:** Richard S. Page, Sean L. McGee, Kevin Eng, Graeme Brown, Sally Beattie, Fiona Collier, Stephen D. Gill

**Affiliations:** 1 Barwon Centre for Orthopaedic Research and Education (B-CORE), Barwon Health, St John of God Hospital and Deakin University, PO Box 281, Geelong, 3220 Australia; 2 School of Medicine, Deakin University, Waurn Ponds, 3216 Australia; 3 Orthopaedic Department, Barwon Health, Geelong, 3220 Australia; 4 Geelong Centre for Emerging Infectious Diseases (GCEID), Geelong, 3220 Australia

**Keywords:** Adhesive capsulitis, Frozen shoulder, Transcriptomics, Biomarkers

## Abstract

**Background:**

Adhesive capsulitis (AC) is a disabling and poorly understood pathological condition of the shoulder joint. The current study aims to increase our understanding of the pathogenesis, diagnosis and clinical outcomes of people with AC by investigating: 1) transcriptome-wide alterations in gene expression of the glenohumeral joint capsule in people with AC compared to people with non-inflammatory shoulder instability (controls); 2) serum and urine biomarkers to better understand diagnosis and staging of AC; and 3) clinical outcomes in people with AC compared to controls 12-months following arthroscopic capsular release or labral repair respectively.

**Methods:**

The study is a prospective multi-centre longitudinal study investigating people undergoing arthroscopic capsulotomy for AC compared to people undergoing arthroscopic stabilization for shoulder instability. Tissue samples collected from the anterior glenohumeral joint capsule during surgery will undergo RNA-seq to determine differences in gene expression between the study groups. Gene Set Enrichment Analysis will be used to further understand the pathogenesis of AC as well as guide serum and urine biomarker analysis. Clinical outcomes regarding pain, function and quality of life will be assessed using the Oxford Shoulder Score, Oxford Shoulder Instability Score, Quick DASH, American Shoulder and Elbow Society Score, EQ-5D-5 L and active shoulder range of movement. Clinical outcomes will be collected pre-operatively and 12-months post-operatively and study groups will be compared for statistically significant differences using linear regression, adjusting for baseline demographic variables.

**Discussion:**

This study will provide much needed information regarding the pathogenesis, diagnosis and staging of AC. It will evaluate clinical outcomes for people undergoing arthroscopic release of AC by comparing this group to people undergoing arthroscopic surgery for shoulder instability.

**Trial registration:**

ACTRN12618000431224, retrospectively registered 26 March 2018.

## Background

Adhesive capsulitis (AC), or ‘frozen shoulder’ is a debilitating pathological condition of the glenohumeral joint, characterised by stiffness, pain and dysfunction [1, 2]. The condition is estimated to affect 2–5% of the general population, most commonly in women and people aged 40 to 60 years [3, 4]. The natural history of AC has been described as self-limiting, with resolution of symptoms within 2 years [5]. However, many people experience prolonged symptoms and functional limitations [6]. Hand et al. [7] found that 41% of 223 patients reported ongoing symptoms at 4 years following symptoms onset, with 6% still reporting severe pain and functional loss.

Diagnosing AC is usually based on clinical findings [6]. The American Shoulder and Elbow Surgeons consensus definition describes AC as “functional restriction of both active and passive shoulder motion for which radiographs of the glenohumeral joint are essentially unremarkable except for the possible presence of osteopenia or calcific tendonitis” [8]. However, in the absence of objective criteria, particularly in the early-stage AC [9], diagnosis can be contentious [10]. Patients usually report the onset of shoulder pain before loss of motion [11, 12]. External rotation is often the first reported movement restriction [1, 13], which is thought to be consequential to contraction of the coracohumeral ligament, one of the essential diagnostic findings in AC [1]. X-rays can be used to rule out other causes of shoulder pain and stiffness [14]. Ultrasound, magnetic resonance imaging (MRI) and magnetic resonance arthrography can detect features suggestive of AC such as capsular thickening, and loss of the inferior axillary pouch and joint volume [14]. Blood tests are usually normal, though cholesterol, triglycerides and C-reactive protein might be elevated in the early stages [10].

The major pathological features of AC are inflamed glenohumeral and subacromial synovium, and thickening and contracture of joint capsule, particularly the rotator interval and coracohumeral ligament [1]. Inflammatory and fibrogenic cells and mediators such cytokines, macrophages and mast cells are elevated in AC capsular tissues compared to controls [15–17]. Inflammatory processes might initiate fibroblast proliferation and fibrotic processes, producing an imbalance between extracellular matrix tissue degradation, remodelling and regeneration; this disequilibrium might lead to fibrosis [1, 18]. Fibroblasts might also transform into myofibroblasts and cause capsular contracture [19], but myofibroblasts, also found in Dupuytrens disease associated with AC [20], have not been found in all AC tissue samples [21]. Neoangiogenesis and neoinnervation also occur in AC; the latter process may explain why AC can be intensely painful [1].

Although the macroscopic and histological features of AC are well described, the underlying pathogenic processes are less understood [18, 22]. In primary idiopathic AC, the aetiology is uncertain [22]. In secondary AC, there may be a history of trauma or surgery, myocardial infarction, type I or type II diabetes mellitus, hypothyroidism, or Parkinson disease [22]. Arkikila et al. [23] found the incidence of AC in people with type I or type II diabetes was 10 and 22% respectively.

The transcriptional profile of AC has received little research attention [12]. Evidence suggest a genetic predisposition towards AC using family history and racial predilection as markers for genetic association [22]. Identification and functional analysis of changes in gene expression associated with AC might help understand its pathogenesis [24]. Cui et al. [24] used RNA-sequencing to investigate the pathogenesis of idiopathic AC by comparing five tissue samples from people with AC to two people with acromioclavicular dislocation patients. One-hundred and eighty-eight differentially expressed genes were identified which encode proteins such as matrix metalloproteinases and cytokines. Hagiwara et al. [21] compared tissue samples from 12 people with idiopathic AC shoulders to18 people with rotator cuff tears and found the expression of 44 genes were significantly different; these genes related to fibrosis, inflammation, chondrogenesis and angiogenesis. Increasing the statistical power for these analyses might reveal new insights in AC pathogenesis and identify new biomarkers for diagnosis and treatment.

### Study aims

The primary aim of the current study is to investigate gene expression alterations in people with primary and secondary AC compared to people with non-inflammatory shoulder instability (controls). Secondary aims are: 1) to interrogate gene expression data to identify potential serum and urine biomarkers that might assist in the early diagnosis and staging of AC; and 2) investigate clinical outcomes in people with AC compared to controls 12-months following arthroscopic capsular release or labral repair respectively.

## Methods

### Study design

**Fig. 1:**
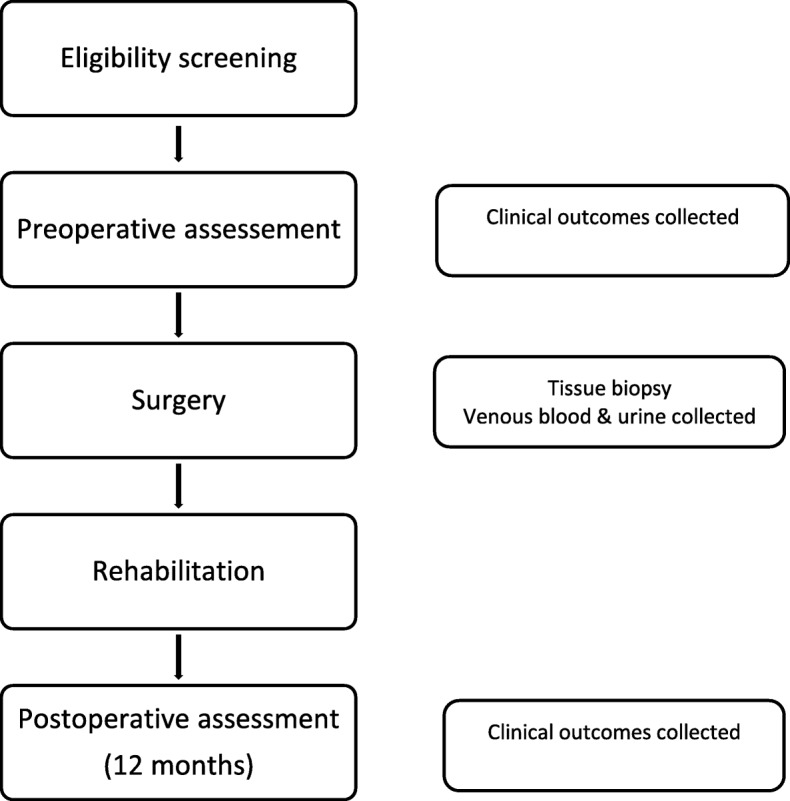
Study flow diagram

This prospective longitudinal multi-centre study investigates the pathophysiology and clinical status of people with AC compared to people with shoulder labral tears and associated instability. See Fig. 1 for an outline of the study.

### Setting

The study will be conducted at two large regional health services in Victoria, Australia: University Hospital Geelong and St John of God Hospital Geelong.

All participants will be recruited by one of three fellowship trained orthopaedic surgeons (RP, KE, GB) who subspecialize in shoulder surgery. Participants will be undergoing elective arthroscopic shoulder surgery for either capsular release for AC or labral repair for shoulder instability. Similar to other studies [25], patients with shoulder instability undergoing arthroscopic stabilization were chosen as the control group; these patients, for whom tissue samples can be taken, are not thought to have primary disease affecting the shoulder capsule [26], but experience significant pain and functional limitations [27].

### Eligibility criteria

Eligible participants have the following characteristics:

1. 18 to 70 years of age
2. Symptoms > 3 months
3. Normal x-ray
4. No evidence of arthropathy or full thickness rotator cuff tear on bloods and MRI
  - MRI studies will be performed on minimum 1.5 T scanners using the sequences: axial T1-weighted, coronal oblique T2-weighted with and without fat saturation, axial and sagittal oblique proton density, and sagittal oblique T2 fat saturation
5. AC group (diagnosis based on consensus definition [8])
  - Primary or secondary AC
  - Loss of shoulder active motion > 30% in all directions compared to unaffected side including at least 50% reduction in external rotation
6. Comparison group
  - Greater than one episode of glenohumeral instability, defined as documented dislocation with labral tear on MRI
  - No episode of instability in eight weeks prior to surgery (to reduce the likelihood of acute post-traumatic inflammatory reaction in the shoulder capsule)
7. Willing, able and mentally competent to provide informed consent (able to read and understand the Patient Information and Consent Form which is written in English language).

People who have the following characteristics are not eligible:

1. Bilateral shoulder pain or reduced motion affecting daily living
2. Prior shoulder surgery

Consecutive patients who fulfill the eligibility criteria will be invited to participate by a study surgeon at the pre-operative assessment. Informed written consent will be obtained as appropriate. Participation in the study is voluntary; no financial incentives will be offered, and recruitment will occur over a 2-year period, commencing in 2016.

### Biosamples

Urine and venous blood samples will be collected immediately before the operation and transported to the Geelong Centre for Emerging Infectious Diseases (GCEID) laboratory for aliquoting and storage in secure − 80 degrees Celsius freezers. Venous blood will undergo the following tests: full blood examination; renal, thyroid and liver function tests; random glucose level; and HbA1C.

### Surgical procedures

One of three fellowship trained orthopaedic shoulder surgeons (RP, KE, GB) will conduct each surgical procedure.

All participants receive general anaesthetic and interscalene block, and will be placed in the beach chair or lateral position consistent with published guidelines [28]. During the arthroscopy, two punch biopsies the size of rice grain will be collected under direct arthroscopic vision from the anterior capsule and rotator interval. Tissue samples are immediately placed in a vial containing RNA*later*™ stabilization solution (ThermoFisher).

In the AC group, each participant’s shoulder will be accessed with three arthroscopic portals and saline arthroscopic fluid at room temperature inserted into the joint space. Biopsies will be taken and rotator interval release conducted via an incision of the anterior capsule with the radiofrequency probe in the 1.00 to 5.30 position. Range of motion will be compared to the unaffected shoulder and a limited posterior capsule release performed by reversing the arthroscopic view if ranges are not equal.

In the instability group, each participant’s shoulder will be evaluated under anesthetic to determine the stability pattern of the glenohumeral joint, with clinical findings considered alongside pre-operative MRI findings. The joint space will be accessed with arthroscopic portals and biopsies collected. The labral tear will be prepared with liberator probes and shaver, and capsulolabral reduction and fixation anteriorly +/− posteriorly will occur with anchors according to the injury pattern.

Postoperative care of all participants, irrespective of group allocation will be according to the study centres’ protocols and care pathways. All participants will stay in hospital overnight. AC group participants will commence physiotherapist-supervised stretching and active-assisted ranging exercises from day 1 post-surgery. Instability group participants will use an immobilizer sling for at least four weeks post-surgery and then commence a rehabilitation program to improve active shoulder stability. Rehabilitation will be monitored for at least three months postoperatively. Incremental increases to range of movement exercises, strengthening and functional restoration will be incorporated into each participant’s individualized program. For the instability group, return to sport will be allowed after six months, pending the participant’s recovery.

### RNA extraction and transcriptomics analysis

Total RNA will be extracted from all samples using a commercially available kit (Qiagen). The quantity and quality of the extracted RNA will be measured by Agilent bioanalyser. Sequencing libraries will be generated from 1 μg of RNA using the Truseq RNA library preparation kit (Illumina) before being converted to complimentary DNA (cDNA). cDNA libraries will then be sequenced (Illumina HiSeq). Raw read quality filtering and adapter trimming will be performed with Trimmomatic before building of the transcriptome index with Spliced Transcripts Alignment to a Reference (STAR) Software [29]. Collation of individual sample counts into a m x n matrix for differential abundance testing will be performed utilising R using t-tests after total-count normalisation. The false discovery rate (FDR) will be determined to control for multiple comparison testing. Furthermore, Gene Set Enrichment Analysis (Broad Institute) will be used to determine pathway-specific alterations in gene expression, which will give new insights into the pathogenesis of AC. Differentially expressed genes will undergo further bioinformatics analysis to identify genes that contain an export sequence motif common to all secreted proteins [30]. In addition, differentially expressed genes encoding proteins that are exported via exosome vesicles will also be identified [31]. Identified candidate genes will be confirmed by quantitative PCR. PCR data will be expressed as mean ± SEM, and differences between groups will be assessed using t-tests and considered statistically significant when *p* < 0.05. Potential biomarker genes that are significantly different between groups will guide analysis of plasma and urine samples from AC and control patients to generate information relating to specific biomarkers.

### Clinical outcomes

Patient reported outcome measures will be collected pre-operatively and 12-months post-operatively and include the Oxford Shoulder Score [32], Oxford Shoulder Instability Score [33], Disabilities of the Arm, Shoulder, and Hand Questionnaire Short Version (Quick DASH) [34], American Shoulder and Elbow Society Score [35], and the EQ-5D-5 L [36]. Standardised data collection will be overseen by the study’s research manager. Active range of movement of the affected shoulder will be assessed by the patient’s surgeon pre-operatively and 12-months postoperatively. To maximise follow up rates, assessment of 12-month outcomes will be aligned with the study centre’s registry research program which routinely collects 12-month postoperative data [37].

Outcome data will be summarised using means with standard deviations or medians with lower and upper quartiles if the data are skewed. Outcomes for AC and control participants will be compared pre-operatively and at 12 months using linear regression, adjusted for potential confounders such as age, employment status and dominant arm. Tests will be considered significant when *p* < 0.05. The limitations of comparing inherently different groups, for example with different pathology and treatment, will be recognised when interpreting the results.

### Demographic data

At the pre-operative assessment, the following information will be collected from each participant: age, sex, employment status, dominant arm, symptom duration, employment status, type of work (e.g. heavy manual), prior treatment (e.g., physiotherapy, corticosteroid injection, hydrodilation), comorbidities and current medications.

### Data management and blinding

Research personnel will keep records of sample collection and processing times, and enter clinical data into REDCap [38], a password-protected electronic data collection and management tool hosted at Barwon Health.

Personnel assessing tissue samples will be blinded to the study group. Functional outcome scores will be collected independent of surgeons; however, surgeons will collect shoulder active range of movement scores.

### Sample size

The majority of the molecular parameters being compared in this study are new in this disease setting and few datasets exist from which to estimate the sample size. Hagiwara et al. [21] found significant differences in gene expression for 33 genes with 12 AC and 18 control samples. To increase the likelihood of detecting differences in gene expression between our groups, we plan to recruit 25 participants per group. This sample size represents a pragmatic decision, based on our expectation of being able to recruit 50 participants over a two-year period, and our desire to increase the statistical power of our study compared to other research.

## Discussion

The current study will fill a knowledge gap and provide much needed empirical evidence regarding the pathogenesis of AC. The results may be used to develop more effective methods to diagnose and treat AC.

## Abbreviations

AC: Adhesive capsulitis
MRI: Magnetic resonance imaging
